# Prevalences and excretion levels of *Lawsonia intracellularis*, *Brachyspira pilosicoli* and *Escherichia coli* F4 and F18 in fecal sock samples from Danish weaner and finisher pig batches and the association with diarrhea

**DOI:** 10.1186/s40813-022-00290-x

**Published:** 2022-10-28

**Authors:** Susanne Leth Musse, Gitte Blach Nielsen, Helle Stege, Nicolai Rosager Weber, Hans Houe

**Affiliations:** 1 MSD Animal Health Nordics, Havneholmen 25, 1561 Copenhagen, Denmark; 2 Section for Production, Nutrition and Health, Department of Veterinary and Animal Sciences, University of Copenhagen, Grønnegårdsvej 2, 1870 Frederiksberg, Denmark; 3 Danish Agriculture and Food Council, Axelborg, Axeltorv 3, 1609 Copenhagen, Denmark; 4 Section for Animal Welfare and Disease Control, Department of Veterinary and Animal Sciences, University of Copenhagen, Grønnegårdsvej 8, 1870 Frederiksberg, Denmark

**Keywords:** Enteric bacteria, Diarrhea, Fecal sock sampling, Batch, Prevalence, Bacterial excretion level, *Lawsonia intracellularis*, *Brachyspira pilosicoli*

## Abstract

**Background:**

Bacterial enteritis in growing pigs is a matter of concern in Danish pig production challenging herd health as well as production economy, and antimicrobial usage. The aim of this observational study using fecal sock samples was to determine the prevalence and excretion level of *Lawsonia intracellularis* (LI), *Brachyspira pilosicoli* (BP), *Escherichia coli F4* (F4) and *F18* (F18) and to investigate associations between prevalence or excretion levels of the bacteria and diarrhea. The study was performed in the late weaner and the early finisher period in herds with a history of diarrhea. Every weaner and finisher herd contributed with one sample each.

**Results:**

In total, 47 weaner and 59 finisher herds were sampled. The overall prevalence and excretion levels (median and range in log(10) copies/gram of feces) were for LI 84.0% (median 6.2; range 3.0–7.7), for BP 45.2% (median 5.6; range 3.0–6.6), for F18 20.8% (median 5.7; range 4.7–7.7), and for F4 4.7% (median 5.5; range 5.2–6.0). In both diarrheic and non-diarrheic samples, the most prevalent bacteria were either LI alone or LI and BP in combination. In general, no association was found between increasing total bacterial excretion levels and diarrhea, but prevalence (*p* = 0.04) and excretion (*p* < 0.01) level of F18 was found to be significantly higher in diarrheic samples. Further, a significant association was found between low LI excretion level and lack of diarrhea in weaner herds (*p* = 0.03). A significant positive correlation was found between excretion levels of LI and BP in diarrheic weaner herd samples (*p* = 0.02).

**Conclusion:**

Enteric pathogens were prevalent in a wide range of bacterial excretion levels in both diarrheic and non-diarrheic samples. Especially LI and BP were frequently found and with a positive correlation between excretion levels. Even in the absence of diarrhea, high prevalence and excretion levels of LI and BP were detected, thus making the status of diarrhea an insufficient tool for assessing the severity of their infections.

## Background

In the Danish pig production, all stakeholders constantly focus to maintain and improve health. Healthy animals are beneficial for animal welfare and production economy. Further, it ensures high food safety with reduced need for antimicrobial usage. Antimicrobial reduction is demanded to accommodate the increasing regulations on antimicrobial usage imposed on the production by political hand [1–4]. In 2020, the Danish pig production accounted for 76% of antimicrobials prescribed for production animals in Denmark, measured in tonnes of active compound [4]. The antimicrobials are mainly used for treating intestinal infections in growing pigs, and for this age group, oral group medication is widely used [5]. To perform group medication in pigs in Denmark, regular use of laboratory diagnostics to confirm the diagnosed bacterial infections is required [6]. A widely chosen form of diagnostic sampling for this purpose is fecal sock sampling as previously described [6–8]. Typically, the samples are analyzed by quantitative Polymerase Chain Reaction (PCR) for *Lawsonia intracellularis* (LI), *Brachyspira pilosicoli* (BP)*, E. coli* fimbria type F4 (F4) and *E. coli* fimbria type F18 (F18). These bacteria are considered to be the most frequent causes of diarrhea or subclinical enteritis influencing welfare, productivity, and antimicrobial use in growing pigs in Denmark [9–12].

In Denmark, LI is regarded as the main intestinal bacterium causing diarrhea in growing pigs later than 2 weeks post weaning. The prevalence and excretion of LI increase with time post weaning, starting typically from around 8–18 weeks of age and peaking around 12 weeks of age [9, 13, 14]. The prevalence and excretion of BP also increase with time post weaning, but instead of declining in appearance during the mid-finisher period, BP may be present throughout the finisher period in infected herds [9]. F4 and 18 are usually detected in the early to mid-weaner period, although sporadic detection can be observed in the late weaner period or even in finisher pigs [7].

Research in diarrheic diseases in pigs has often focused on single pathogens and has less frequently investigated the occurrence of co-infections, with the potential risk of misinterpreting the clinical relevance of mixed infections. In a Danish study in diarrheic weaner herds, 45% of the cases had a combination of LI, BP, F4 and F18 [8]. In another Danish study from 1998, bacterial co-infections were found more frequently in diarrheic than in non-diarrheic grower herds [15], but this was not confirmed in a more recent study in weaner herds from 2015 [11]. Several prevalence studies imply a correlation between LI and BP as a high concomitant prevalence of LI in samples positive to BP has been found [10, 15–18]. In addition to prevalence, total bacterial excretion level can be of clinical relevance for the determination of the severity of diarrhea [19]. A classification of diarrheic outbreaks in weaner herds as either low-pathogen diarrhea or high-pathogen diarrhea by the total bacterial excretion level of F4, F18, LI, and BP as a diagnostic tool to determine the occurrence of bacterial enteritis has been recommended [20].

Fecal sock samples collected from pig herd batches both with and without outbreak of diarrhea can detect differences in prevalence and excretion level of bacteria. This will increase the ability to choose optimal strategies for treatment and prevention of diarrhea not just beneficial to animal welfare and productivity [21], but also to further reduce antimicrobial usage. Using fecal sock samples collected at batch level in the late weaner period and in the early finisher period, the objectives of this study were to:

1. Determine the prevalence and bacterial excretion level of LI, BP, F4 and F18 in samples collected from commercial pig herds with or without diarrheic outbreak.
2. Investigate potential associations between diarrhea and either the prevalence or excretion level of bacteria.

To examine objective 2, the following 3 hypothesis were established:

- H1: There is an association between bacterial prevalence or bacterial excretion level and diarrhea for each of the four different bacteria as well as for the total bacterial excretion level.
- H2: Diarrhea occurs more frequently in mixed bacterial infections.
- H3: There is a correlation between different bacteria in prevalence or in excretion level.

## Results

The study was conducted in a total of 106 herds of which 47 were weaner herds and 59 were finisher herds. The main part of the herds was managed as most other commercial herds in Denmark, whereas one weaner herd and one finisher herd were OUA herds (“reared without use of antimicrobials”) and another weaner herd and another finisher herd were organic. Additionally, four weaner herds and nine finisher herds herds were initially referred to the study but were excluded due to lack of sock sampling or samples collected from batches not being within the period of interest. The yearly production from each weaner herd was in a range of 7.500–35.000 pigs and in each finisher herd in a range of 2.500–32.000 pigs. With the given samples size in the least represented group of 21 diarrheic weaner herds and with samples having a LI herd prevalence > 0.8, the herd prevalence could be determined with an allowable error L = 0.17 [22].

Of the 47 weaner herds samples, 26 samples were collected from non-diarrheic batches and 21 samples were collected from diarrheic batches. Of the 59 finisher herd samples, 33 samples were collected from non-diarrheic batches and 26 samples were collected from diarrheic batches.

Due to the study design, most of the non-diarrheic samples were collected in the last part of the period of interest in both weaner and finisher herds.

The overall prevalence and excretion levels of bacteria are presented in Table 1. LI was the most frequently found bacteria with a prevalence of more than 80% regardless of the age group. BP was more prevalent in finisher herds than in weaner herds, whereas the opposite applied for F18. F4 was found in just five samples and will only be given further attention as part of the total sum of prevalent bacteria and in total excretion level of bacteria.

**Table 1:** Prevalence of *Lawsonia intracellularis*, *Brachyspira pilosicoli*, *Escherichia coli* F4 and *Escherichia coli* F18 and their excretion levels in fecal sock samples from weaner herds and finisher herds

Sample group	Both herd types		Weaner herd		Finisher herd	
Total number of sock samples	106		47		59	
	n, (prevalence %)	Excretion level^a^	n, (prevalence %)	Excretion level^a^	n, (prevalence %)	Excretion level^a^
*Lawsonia intracellularis*	89 (84.0%)	6.2; 3.0–7.7	39 (83.0%)	6.2; 3.0–7.7	50 (84.7%)	6.1; 3.0–7.7
*Brachyspira pilosicoli*	48 (45.3%)	5.6: 3.0–6.6	18 (38.3%)	5.7; 3.0–6.4	30 (50.8%)	5.5; 3.0–6.6
*Escherichia coli F4*	5 (4.7%)	5.6; 5.2–6.0	3 (6.4%)	5.6; 5.5–6.0	2 (3.4%)	5.4; 5.2–5.6
*Escherichia coli F18*	22 (20.8%)	5.7; 4.7–7.8	13 (27.8%)	5.9; 4.7–7.8	9 (15.3%)	5.5; 4.9–6.3

^a^Median; detection range, in positive samples in log(10) copies/gram of feces

The distribution of bacterial detection in the sock samples is presented in Table 2.

**Table 2:** Detection of *Lawsonia intracellularis*, *Brachyspira pilosicoli*, *Escherichia coli* F4 and *Escherichia coli* F18 (none, single and multiple detection) in all fecal sock samples (both weaner and finisher herd samples)

Status of diarrhea^b^	Number of samples^c^	Prevalence^d^	*Lawsonia intracellularis*^a^	*Brachyspira pilosicoli*^a^	*Escherichia coli* F4^a^	*Escherichia coli* F18^a^
0	22	37.3%	X	0	0	0
0	19	32.2%	X	X	0	0
0	5	8.5%	X	X	0	X
0	5	8.5%	0	0	0	0
0	4	6.8%	0	X	0	0
0	2	3.4%	X	0	0	X
0	1	1.7%	0	0	0	X
0	1	1.7%	X	0	X	0
1	13	27.7%	X	X	0	0
1	12	25.5%	X	0	0	0
1	7	14.9%	X	0	0	X
1	5	10.6%	0	0	0	0
1	5	10.6%	X	X	0	X
1	1	2.1%	X	0	X	0
1	1	2.1%	X	0	X	X
1	1	2.1%	X	X	X	0
1	1	2.1%	0	0	X	X
1	1	2.1%	0	X	0	0

^a^0**—**absence of the pathogen; X**—**presence of the pathogen (grey background)

^b^0**—**absence of diarrhea; 1**—**presence of diarrhea

^c^Total number of samples in category

^d^Prevalence of bacteria combination

The most prevalent detection of pathogens was either LI alone or a combination of LI and BP in both diarrheic and non-diarrheic samples. Both diarrheic and non-diarrheic samples were without detection of any of the bacteria investigated with a prevalence of 8.5% and 10.6%, respectively.

### Association between prevalence of bacteria and diarrhea

Associations between prevalence of bacteria and diarrheic status are presented in Table 3. LI had similar prevalences at 81.0–88.5% in all four groups. Despite displaying a greater numerical difference in the prevalence of BP in diarrheic versus non-diarrheic samples for both age groups, no significant association was found between prevalence of BP and diarrhea. A tendency of association between prevalence of F18 and diarrhea was found when assessing samples from both age groups (*p* = 0.05; OR 2.7; CI 95% 1.0–7.2) implying that the odds of a sample being collected at an outbreak of diarrhea was 2.7 times higher if the sample was positive to F18. This tendency was not detected when assessing weaner herd samples and finisher herd samples separately.

**Table 3:** Associations between prevalence of bacteria in weaner and finisher herd sock samples and diarrhea

	Weaner herd				Finisher herd			
	Diarrhea, n (%)	No diarrhea, n (%)	OR^a^	*P *value^a^	Diarrhea, n (%)	No diarrhea, n (%)	OR^a^	*P *value^a^
Total number of herds and samples	21	26			26	33		
*Lawsonia intracellularis*	17 (81.0%)	22 (84.6%)	OR = 0.78	*p* = 1 (CI = 0.12–4.83)	23 (88.5%)	27 (81.8%)	OR = 1.69	*p* = 0.72(CI = 0.32–11.61)
*Brachyspira pilosicoli*	9 (42.9%)	9 (34.6%)	OR = 1.41	*p* = 0.76 (CI = 0.34–4.62)	11 (42.3%)	19 (57.6%)	OR = 0.54	*p* = 0.30 (CI = 0.19–1.53)
*Escherichia coli F4*	2 (9.5%)	1 (3.8%)			2 (7.7%)	0 (0.0%)		
*Escherichia coli F18*	8 (38.1%)	5 (19.2%)	OR = 2.58	*p* = 0.20 (CI = 0.08–9.67)	6 (23.1%)	3 (9.1%)	OR = 3.00	*p* = 0.16 (CI = 0.67–13.4)

^a^Fisher’s exact probability test

No significant association was found between number of different bacterial species present and diarrhea.

In the multivariable analysis for association between prevalence of bacteria and diarrhea (Model 1), only prevalence of F18 proved to be of significance (*p* = 0.04) when including all samples confirming the OR given by the Fishers exact probability test. Therefore, the final Model 1 with the lowest AIC included only the effect of prevalence of F18: *Diarrhea* ~ *F18 prevalence.* None of the other variables or their interaction were significant neither when assessing all samples, weaner herd samples or finisher herd samples separately nor when excluding F18 due to small number of positive samples.

### Association between bacterial excretion level and diarrhea

Total bacterial excretion level was divided into four ordered groups by cut-off values as given in Appendix Table 4. Based on Model 2, no significant association between total excretion level and diarrhea was found (*p* = 0.09).

**Table 4:** Categorized total bacterium excretion as sum of bacterial excretion of *Lawsonia intracellularis*, *Brachyspira pilosicoli*, *Escherichia coli* F4 and *Escherichia coli* F18 and cut-off values

Variable	Level	Cut-off values^a^	Number of samples (diarrheic:non-diarrheic)
Weaner herd	Negative Low Medium High	0 (0;5.9) (6.0;6.9) (7.0;7.8)^b^	6 (3:3) 15 (2:13) 13 (8:5) 13 (8:5)
Finisher herd	Negative Low Medium High	0 (0;5.9) (6.0;6.9) (7.0;7.7)^b^	4 (2:2) 23 (9:14) 20 (11:9) 12 (4:8)

^a^log(10) copies/gram of feces

^b^Upper limit equals maximum observed value in data set

The excretion level of each bacterium was divided into ordered groups by cut-off values as given in Appendix Table 5. This was done to evaluate association between the excretion level of each bacterium and diarrhea and further to evaluate interactions between bacterial excretion levels of different bacteria and the association to diarrhea. Based on model 3, the end-model with the lowest AIC was: *Diarrhea* ~ *F18 excretion level* + *age group* + LI excretion level *age group + BP excretion level *age group, with a significant association between increasing F18 excretion level and diarrhea (*p* < 0.01) and between diarrhea and the interaction between LI excretion level and age group (weaner, *p* = 0.03). Further, a tendency of association between diarrhea and the interaction between BP excretion level and age group was found (weaner, *p* = 0.05). In contrast to BP and F18, increasing excretion level of LI was, overall, not associated with diarrhea in weaner herd samples, since the association was found between the lowest LI excretion level and lack of diarrhea (*p* = 0.01). All two-way interactions between individual bacteria were found non-significant.

**Table 5:** Categorized bacterial excretion level of *Lawsonia intracellularis*, *Brachyspira pilosicoli* and *Escherichia coli* F18 and cut-off values

Variable	Level	Cut-off values^a^	Number of samples (diarreic:non-diarrheic)
*Lawsonia intracellularis* (Weaner samples)	Negative Low Medium High	0 (0;5.0) (5.1;6.6) (6.7;7.7)^b^	8 (4:4) 11 (1:10) 15 (8:7) 13 (8:5)
*Lawsonia intracellularis* (Finisher samples)	Negative Low Medium High	0 (0;5.0) (5.1;6.6) (6.7;7.8)^b^	9 (3:6) 19 (8:11) 13 (8:5) 18 (7:11)
*Brachyspira pilosicoli* (Weaner samples)	Negative Low Medium High	0 (0;4.9) (5.0;5.8) (5.9;6.5)^b^	29 (12:17) 5 (1:4) 7 (3:4) 6 (1:6)
*Brachyspira pilosicoli* (Finisher samples)	Negative Low Medium High	0 (0;4.9) (5.0;5.8) (5.9;6.7)^b^	29 (15:14) 11 (6:6) 10 (2:8) 9 (4:5)
*E. coli F18* (all samples)	Negative Low Medium High	0 (0;5.3) (5.4;5.9) (6.0;7.8)^b^	84 (33:51) 7 (2:5) 7 (5:2) 8 (7:1)

F4 is not shown due to the low number of positive samples

^a^log(10) copies/gram of feces

^b^Upper limit equals maximum observed value in data set

### Correlation of simultaneous detection of bacteria

For both diarrheic and non-diarrheic samples, in 89.6% of the BP positive samples also LI was detected, whereas only 48.3% of LI positive samples were also BP positive. When testing weaner herd samples, a correlation between prevalence of LI and BP was present (*p* = 0.02;* Conf. interval: 1.23-inf*.). No correlations were detected when testing other age groups or simultaneous detection of other bacteria.

**Fig. 1:**
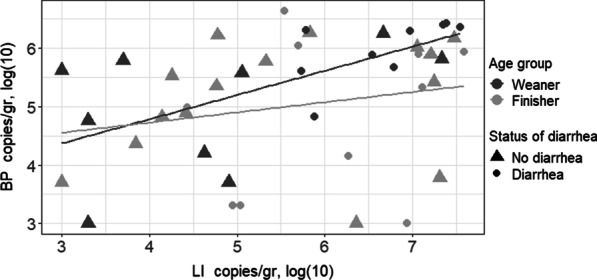
Correlation between *Lawsonia intracellularis* and *Brachyspira pilosicoli* by age group and status of diarrhea in samples positive to the two bacteria. Linear regression lines are added for all Weaner herd samples (*p* < 0.01) (dark grey) and all finisher herd samples (*p* = 0.22) (light grey)

For the 43 samples positive to both LI and BP, a positive correlation was found between LI and BP excretion levels (*p* < 0.01; rho = 0.46). However, when adjusting for age group and status of diarrhea, this correlation was only significant in diarrheic weaner herd samples (9 samples; *p* = 0.02; rho = 0.73) as shown in Fig. 1. No significant correlation was found in finisher herd samples (25 samples; *p = *0.22; rho = 0.25) or in non-diarrheic weaner herd samples (9 samples; *p* = 0.21; rho = 0.46). No correlations between excretion level of either LI and F18 or BP and F18 were found.

## Discussion

This observational study included 106 herds, which is a considerable number of herds compared to previous studies [11, 13, 15, 16, 18–20]. The samples in this study were collected as one sample from each herd from a single batch. Despite this being a well-documented [8, 11] and often used method and a valuable tool [23] for diagnostics in commercial pig herds, the samples do not reflect any within herd fluctuations [9] but only reflects the situation in the given batch at the given day. An information bias due to misclassification could be the evaluation of diarrhea and the need for antimicrobial group treatment, which was done subjectively by the respective herd manager based on instructions given by their herd veterinarians. The sampling was performed by attending all pens in the batch in concern, meaning that in a diarrheic batch also pens without diarrhea were included in the sample and vice versa. This caused a possible risk of falsely reduced or elevated bacterial excretion levels detected in the sock samples but was in accordance with published instructions for sock sampling [8, 12].

The detection of LI being the most frequently occurring bacteria followed by BP, F18 and finally F4 agreed with previous findings for the age group of late weaners and early finishers [9, 10, 23]. The detection of LI in more than 80% of the fecal sock samples was higher than in other recent Danish studies assessing pooled samples, where a prevalence of 40–69% was described [7, 11, 17]. The higher prevalence found in this study could be due to the selection of herds explicitly having a history of diarrheic outbreaks, with previous diagnostic findings supporting LI as a cause of diarrhea and to the study design sampling only from the age group typically at risk of LI infection [13]. The prevalence of BP (45.3%) and F18 (20.2%) were in accordance with previous Danish studies, where BP and F18 were detected in 32–50% and 10–40% of pens sampled, respectively [7, 9, 17].

A tendency between prevalence of F18 and diarrhea was found assessing all samples (*p* = 0.05) but no associations to prevalence of any of the other pathogens were found. Possibly due to the sparse number of samples positive to F18 (22 samples in total), the tendency of F18 disappeared when assessing the age groups separately. This was in alignment with a previous study describing no association between prevalence of F18 and diarrheic events (*p* = 0.06) when monitoring 10 herds by repeated sampling at pen level in the age group form weaning to slaughter [9]. Still, the positive association found between categorized excretion levels of F18 and diarrhea indicated F18 as a potential factor for the occurrence of diarrhea, which is supported by other studies [24].The bacterial excretion levels ranged widely from no detection to massive excretion. In this study, cut-off values dividing the samples into categorized bacterial excretion levels were created to ensure a fairly even distribution of samples in each category. The categorization was made as a best match for the purpose of testing these as explanatory variables. Therefore, our levels were not necessarily in accordance with previously identified levels associated with pathological lesions or reduced productivity. However, for bacteria like LI with a known association between quantities and impact of the infection, the categories medium and high (≥ 5.1 copies/gram feces) in this study were levels previously identified as having a negative impact on productivity [25–29].

No association was found between increased total bacterial excretion levels and diarrhea which agrees with another Danish study [11]. This was the case even though a positive association between increasing bacterial excretion level of F18 and diarrhea was detected. For LI, however, increasing excretion levels were not associated with an increased risk of diarrhea and this is consistent with previous findings [11, 26, 30, 31].

Levels of LI shed in the feces can be used as an indication of the severity of the infection [9, 27] and levels of ≥ 4.8 log(10) bacteria/gram of feces have been found to be associated with proliferative histological lesions in the intestine [26]. In 60.4% of our herds, in both diarrheic and non-diarrheic samples, the LI excretion level exceeded this level. Therefore, in this study, assessing the status of diarrhea in batches of pigs was an inadequate tool for assessing the severity of a bacterial infection as also suggested by other Danish studies [11, 27]. This applied both to weaner pigs, with a significant association between low LI excretion level and less diarrhea, and to finisher pigs where no associations between bacterial excretion level and diarrhea were found at all.

An association between prevalence of LI and BP was present, since almost all BP positive samples were also LI positive, whereas only about half of the LI positive samples were also BP positive, which corresponds to previous findings [10, 15–18]. Further, a correlation between LI and BP excretion levels was found. The correlation was significant in the late weaner period, corresponding to an introduction and onset of infection of both LI and BP.

In agreement with another study [11], simultaneous detection of increasing number of bacterial species did not increase the likelihood of diarrhea and occurrence of diarrhea was a matter of concern also in apathogenic samples. In this study, no pathogens were detected in 10.6% of the diarrheic samples. This corresponds to previous findings [8] and supports that diarrhea can be of non-pathogenic aetiologic origin. Therefore, up to 10% of the batches where an outbreak of treatment-requiring diarrhea was assessed, the use of antimicrobials could be questioned due to the likely non-bacterial etiology. However, the test sensitivity could be less than 100% and the diarrhea could be caused by other pathogens than the ones tested. The risk of the affected batch suffering from *Brachyspira hyodysenteria* or *Salmonella spp.* not detected by the laboratory analysis or from Porcine Circovirus type 2 was considered small, though, due to the inclusion criteria, the Danish SPF-system (85% of the study herds were declared free of *Brachyspira hyodysenteria* or purchased pigs declared free of *Brachyspira hyodysenteria*) and the Danish salmonella surveillance program (all study herds in surveillance level 1 [32]). Just as diarrheic samples without detection of bacteria are of relevance, non-diarrheic samples with bacteria detection are of concern. High-pathogenic diarrhea has been defined as a diarrheic outbreak with a total bacterial excretion level of LI, BP, F4 and F18 ≥ 4.54 log(10) copies/gram feces in a pooled fecal sample [20]. In our study, 77 and 85% non-diarrheic weaner and finisher herd samples, respectively (data not shown), had a total bacterial excretion exceeding this level and could be defined as high-pathogenic even though being collected from batches evaluated without diarrhea These samples from subclinical yet infected batches indicate that interventions by either prevention or treatment could have been of relevance in the affected batch to ensure intestinal health and high productivity. Despite the surge for reduction of antimicrobial usage which could be of relevance in some of the batches sampled, it is of equal importance to assure that subclinical but still costly and welfare compromising infections are not overlooked.

## Conclusion

In this study, the prevalence of LI above 80% was remarkably high in both late weaner period and early finisher period, regardless of diarrhea. The prevalence of F4 (4.7%) was low and prevalence of F18 (20.8%) and BP (45.3%) were similar to previous studies. The bacterial excretion levels of the four bacteria in the sock samples were in a wide range from no detection to massive excretion.

No association was found between bacterial prevalence of LI, BP or F18 and diarrhea.

A positive association was found between bacterial excretion level of F18 and diarrhea and for weaners herds, an association was found between low LI excretion level and non-diarrheic samples. No association was detected between total bacterial excretion level and diarrhea. Detection of increasing numbers of bacterial species of LI, BP, F4 and F18 were not observed more frequently in diarrheic samples than in non-diarrheic samples. A correlation between prevalence of LI and BP was observed as well as a positive correlation between LI and BP excretion levels in weaner herd samples.

In conclusion, based on data from pooled floor samples, F18 was detected in a higher excretion level in diarrheic samples than in non-diarrheic samples. LI and BP were prevalent and even in high excretion level also in samples without diarrhea, thus making the status of diarrhea an insufficient tool for assessing the severity of their infections. BP was more likely to be detected in samples also positive to LI and if simultaneously detected, the two bacteria appeared to potentiate each other’s excretion levels.

## Methods

### Study design

This observational, cross-sectional study was part of a study focusing on the impact of LI, here referred to as “vaccination study”. It was performed in Danish commercial weaner and finisher herds with a history of outbreaks of diarrhea requiring antimicrobial group treatment. All pigs were routinely vaccinated against Porcine Circovirus type 2 at weaning. In the study herds, transfer from weaner to finisher herd was performed at approximately 30 kg equal to seven-eight weeks post weaning, and new batches of pigs entered the herds on a weekly basis. The period of interest were batches five, six and seven weeks post weaning in the weaner herds and batches one, two, three, and four weeks post entry in the finisher herds. Each herd was observed for about two weeks. During this observation period outbreaks of diarrhea requiring antimicrobial group treatment according to normal herd routines were detected by the herd manager or the herd veterinarian. When a diarrheic outbreak was observed one fecal sock sample was collected from the batch in concern prior to antimicrobial treatment. At sampling, all pens of the batch were sampled by one fecal sock sampling as previously described [8]. If no batches experienced an outbreak of diarrhea in the observation period, a fecal sock sample was collected from a batch at a fixed time point. The fixed time point was seven weeks post weaning in weaner herds and three-four weeks post entry in finisher herds, respectively. Every weaner herd and every finisher herd contributed to the study by one sample each. Fecal sock sample kits containing all needed material for the sampling and shipment were supplied by Dianova, National Veterinary Institute, Denmark.

### Sample size

Given that this study was part of a study focusing on the impact of LI vaccination, the sample size was based on assumptions for this bacterium concerning its effect on average daily weight gain (ADG) [33]. Based on identifying a difference in ADG of 40 g/day between vaccinated and non-vaccinated groups and using a standard deviation of 52 g between herds [34] the sample size was estimated to be 27 herds in each group [22]. Assuming that some herds would be withdrawn during the study, the study was aiming at including 40–50 herds for both weaner and finisher herds each.

In the current study the main aim was to determine prevalences. With a LI-herd prevalence of 0.5 (giving the maximum sample size) and accepting the allowable error to be 0.2 of the confidence level of 0.95, 24 herds would be needed in each group (diarrheic or non-diarrheic) for each age group [22].

### Selection of herds

According to the vaccination study protocol, the herds were included in the study based on a herd history of outbreaks of diarrhea, an antimicrobial treatment rate against diarrhea of minimum 25% of the pigs, diagnostic findings supporting LI as a cause of diarrhea, and preferably with a production of no more than approximately 30.000 pigs/year. Herds were identified in close cooperation with local herd veterinarians. Sampling was conducted between November 2019 and April 2020. To minimize the clustering effect of herd complexes, no multisite production contributed with more than a maximum of 3 weaner sites and 3 finisher sites.

### Testing of sampling material

Every fecal sock sample was labelled according to date of sampling, weeks post entry, and status concerning diarrhea. Samples were shipped to the the National Veterinary Institute, DTU, Technical University of Denmark, prepared as previously described [35], and analyzed using a high-throughput real-time PCR system to determine the prevalence and bacterial excretion levels of LI, BP, F4 and F18 [36]. For all four bacteria, samples were considered positive when the sampling result was above the lower detection limit of 3 log(10) copies/gram feces.

### Statistical analysis

The individual bacterial counts of the four bacteria in the sock samples as well as the total bacterial count being the sum of the four individual bacterial counts were logarithmically transformed (log(10)), and bacterial excretion levels were analyzed as continuous data. Analysis was performed on both the total set of samples, and on weaner- and finisher herd samples separately. In the multivariable analysis, backwards elimination to lowest possible value in Akaikes Information Criterion (AIC) was used to find variables of significance in final models. For all statistical tests, a value of *p* < 0.05 was considered significant. Statistical analysis was performed using R version 4.1.2 [37].

#### Association between prevalence of bacteria and diarrhea

To evaluate associations between bacterial prevalence and diarrhea, data was dichotomized, and Fisher’s exact probability test was used due to sparse numbers of observations in some groups. Chi-square test was used to evaluate the association between bacterial detection with increasing number of different bacterial species present and diarrhea. Logistic analysis was used for multivariable analysis, including prevalence of each bacterium and their interaction, as given in Model 1.

- Model 1: Diarrhea ~ LI prevalence + BP prevalence + F18 prevalence + LIprev*BPprev + LIprev*F18prev + BPprev*F18prev.

#### Association between bacterial excretion level and diarrhea

To test the associations between bacterial excretion level and diarrhea and further to be able to include interactions between the different bacteria, bacterial excretion levels were categorized into negative, low, medium, and high levels. Cut-off values were determined to get as far as possible an evendistribution of samples in each category of low, medium and high. Logistic analysis evaluated the association between total bacterial excretion level and diarrhea as given in Model 2. All possible interactions were included into Model 3 testing association between individual bacterial excretion levels, interactions between bacterial excretion level, and diarrhea.

- Model 2: Diarrhea ~ total excretion level + age group + total excretion level *age group.
- Model 3: *Diarrhea* ~ *LI excretion level* + *BP excretion level* + *F18 excretion level* + *age group* + LI excretion level *age group + BP excretion level *age group + F18 excretion level *age group + LI excretion level *BP excretion level + LI excretion level *F18 excretion level + BP excretion level *F18 excretion level + LI excretion level *BP excretion level *age group + LI excretion level *F18 excretion level *age group + BP excretion level *F18 excretion level *age group.

#### Correlation between bacteria in prevalence and in excretion level

Fisher’s exact probability test was used to evaluate simultaneous detection of different bacteria species and Spearman’s rank correlation test was used for testing correlation of bacterial excretion levels.

## Data Availability

The dataset used and/or analyzed during the current study and supporting the conclusions of this article are available from the first author on reasonable request.

## Appendix

See Tables 4 and 5.

## Abbreviations

LI: *Lawsonia intracellularis*
BP: *Brachyspira pilosicoli*
F4: *Escherichia colin F4*
F18: *Escherichia coli F18*
PCR: Polymerase chain reaction
ADG: Average daily gain
AIC: Akaikes information criterion
OUA: Production concept of pigs reared without use of antimicrobials
